# Prognostic nutritional index predicts short-term outcomes after liver resection for hepatocellular carcinoma within the Milan criteria

**DOI:** 10.18632/oncotarget.13151

**Published:** 2016-11-07

**Authors:** Mengyun Ke, Tao Xu, Na Li, Yifan Ren, Aihua Shi, Yi Lv, Haiqi He

**Affiliations:** ^1^ Research Institute of Advanced Surgical Techniques and Engineering, Regenerative Medicine and Surgery Engineering Research Center of Shaanxi Province, The First Affiliated Hospital of Xi'an Jiaotong University, Xi'an, Shaanxi, China; ^2^ Department of Hepatobiliary Surgery, Provincial Hospital Affiliated to Shandong University, Jinan, Shandong, China; ^3^ Department of Laboratory Medicine, The First Affiliated Hospital of Xi'an Jiaotong University, Xi'an, Shaanxi, China; ^4^ Department of Hepatobiliary Surgery, The First Affiliated Hospital of Xi'an Jiaotong University, Xi'an, Shaanxi, China; ^5^ Department of thoracic surgery, The First Affiliated Hospital of Xi'an Jiaotong University, Xi'an, Shaanxi, China

**Keywords:** hepatocellular carcinoma, hepatectomy, postoperative complications, prognostic nutritional index

## Abstract

**Background:**

The prognostic nutritional index (PNI) is calculated based on the serum albumin concentration and the total lymphocyte count. The aim of this study was to investigate the prognostic ability of the PNI for postoperative complications after liver resection to treat hepatocellular carcinoma (HCC) within the Milan criteria.

**Results:**

Postoperative complications were observed in 166 (44.6%) patients. The optimal cutoff value of the PNI was set at 45.6 for postoperative complications. Patients in the PNI-low (PNI < 45.6) group were more likely to have postoperative complications, more blood loss, a longer surgery time and a longer hospital stay than patients in the PNI-high group (PNI > 45.6). Our regression analysis demonstrated that the preoperative PNI and albumin-bilirubin (ALBI) score were significantly associated with postoperative complications (Pearson correlation coefficient, -0.865, *p* < 0.001). The multivariate analysis revealed that the PNI was an independent predictor of postoperative complications.

**Materials and Methods:**

Three-hundred and seventy-two patients who underwent partial hepatectomy for HCC from 2003 to 2014 were identified. The cutoff value of the PNI was determined by a receiver operating characteristic (ROC) curve analysis. Univariate and multivariate analyses were performed to identify clinicopathological features associated with postoperative complications.

**Conclusion:**

The PNI may be a significant prognostic factor for evaluating short-term outcomes of patients with HCC after partial hepatectomy.

## INTRODUCTION

Hepatocellular carcinoma (HCC) is one of the most common cancer and the third leading cause of cancer-relateded death around the world [1, 2]. Partial hepatectomy is one of the most important methods to treat HCC patients. As surgical techniques and perioperative care have advanced, the post-operative outcomes have improved significantly over the past several decades [3, 4]. Nevertheless, the postoperative morbidity remains high. Therefore, the accurate identification of patients with high risks of postoperative complications plays a vital role in improving patient survival.

The patient's nutritional status is tightly related to the function of immune system, the changes of inflammatory response, and the wound healing process [5]. Cancer cachexia is common in HCC patients. The previous study has demonstrated that preoperative enteral nutrition could improve short-term surgical outcomes of HCC patients [6]. However, the published work did not raise an exact nutrition index to predict the surgical outcome of liver resection. Prognostic nutritional index (PNI), which is calculated based on the concentration of serum albumin and the count of total lymphocyte, was demonstrated to be an effective prognostic factor in several cancers [7]. In this study, we used the PNI to conduct a retrospective cohort study of HCC patients within the Milan criteria undergoing surgical resection. We demonstrated that the PNI is a significant predictor of the short-term postoperative outcomes of patients with HCC.

## RESULTS

### Patient characteristics

The baseline characteristics of the population in this study are presented in Table 1. The 372 HCC patients consisted of 298 males and 74 females. The average (±SD) age was 51.74 ± 10.87 years, and 298 (80.11%) patients were infected with the hepatitis B virus. On pathology, the mean maximum tumor size was 3.53 ± 1.04 cm, and 322 (86.6 %) patients had solitary tumors.

**Table 1:** Clinicopathological characteristics of the patients

Characteristics	n = 372
Gender Male vs Female	149/37
Age (y), mean±SD	51.74± 10.87
Hepatitis B virus	298
ASA grading	22/228/116/6
TBIL (μmol/L)	27.28± 54.00
AST (U/L), mean±SD	50.56± 51.15
ALT (U/L), mean±SD	52.59± 55.52
ALB (g/l), mean±SD	39.25± 5.79
PLT (10^9^/L), mean±SD	123.37 63.46
WBC (10^9^/L), mean±SD	5.25± 2.52
RBC (10^12^/L), mean±SD	4.22± 0.60
Hb (g/l), mean±SD	130.94± 20.97
PT (s), mean±SD	13.51± 1.37
APTT (s), mean±SD	37.61± 7.12
INR, mean±SD	1.09± 0.12
Number of tumours	
Single	322
Multiple	50
Tumor size (cm), mean±SD	3.53± 1.04
Blood loss (mL)	625.40± 669.22
Surgery time (min)	202.98± 74.99
Pringles maneuver time (min)	46.55± 6.91

Abbreviations: ASA, American Society of Anesthesiologists; TBIL, total bilirubin; AST, aspartate aminotransferase; ALT, alanine aminotransferase; ALB, albumin; PLT, platelet count; WBC, white blood cell count; RBC, red blood cell count; Hb, hemoglobin; PT, prothrombin time; APTT, activated partial thromboplastin time; INR, international normalized ratio.

### ROC analysis

**Figure 1:**
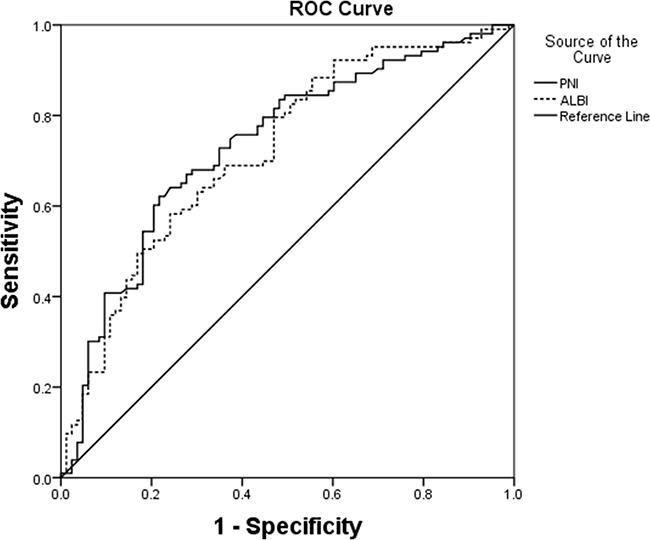
ROC curves for PNI in relation to postoperative complications AUROC was 0.733 (95% confidence interval, 0.681–0.784; *p* < 0.001) for PNI. The calculated cutoff value for PNI was 45.6, with a sensitivity of 69.6% and a specificity of 61.5% in the prediction of complications.

**Figure 2:**
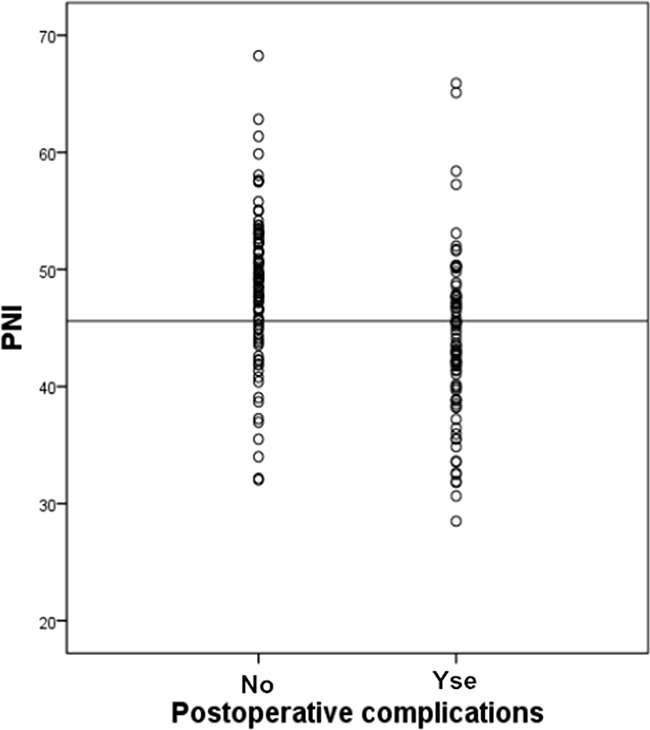
Incidence of postoperative complications following liver resection according to PNI The reference line indicates the PNI of 45.6 is associated with a significant risk of postoperative complications (Student *t* tests; *p* < 0.001).

Postoperative complications are shown in Table 2. There were no deaths within 30 or 90 days. The incidence of complications was 44.6% (166/372), including 140 minor complications and 26 major complications. The diagnostic potential of the PNI in detecting postoperative complication risk is shown in Figure 1. The ROC curve analysis revealed that a PNI value of 45.6 identified patients at significant risk for postoperative complications following liver resection with a sensitivity of 69.6% and a specificity of 61.5%. The area under the ROC curve (AUROC) was 0.733 (95% confidence interval, 0.681–0.784; *p* < 0.001). Additionally, the PNI was comparable to the ALBI score (0.721, 95% confidence interval, 0.648–0.795; *p* < 0.001) in predicting postoperative outcomes. The distribution of PNI values according to the occurrence of complications is shown in Figure 2. The mean PNI value was much lower in patients with postoperative complications compared to those without complications (48.75 (6.10) vs 43.83 (6.92); independent sample t test, *p* < 0.001).

**Table 2:** Postoperative complications according to the modified Clavien classification

Grading of complications	Complication No.	Details of complications
Minor complications	140	
Grade 1	20	Ascites or Hypoproteinemia,12; Fat liquefaction, 2; Hemorrhage, 2; Pneumonia, 2; Hypertension, 2;
Grade 2	120	Ascites, Pleural effusionor or Hypoproteinemia,78 Pneumonia, 18; Perihepatic hydrops, 2; Arrhythmia, 4 Partial ileus, 2 pyelonephritis, 2; Bile leakage, 4; Hemorrhage, 6; Hypokalemia, 2; gastroenteric stress ulcer, 2;
Major complications	26	
Grade 3a	20	Respiratory failure, 2; Renal failure, 2; Wound infection, 1; Bile leakage, 1; Ascites or Pleural effusion requiring drainage, 10; Hemorrhage, 2; Pneumonia, 2;
Grade 3b	2	Hemorrhage, 2
Grade 4a	2	Liver dysfunction, 2;
Grade 4b	0	_
Grade 5	2	Liver failure, 1; Death, 1;
Total	166	

### The associations between PNI and clinicopathological parameters

The associations between the clinicopathological parameters and the PNI are shown in Table 3. The results demonstrate that the higher PNI group had a better hepatic function index (AST (aspartate aminotransferase), TBIL (total bilirubin), albumin) and parameters of coagulation function (PT (prothrombin time), APTT (activated partial thromboplastin time), INR (international normalized ratio)) than the lower PNI group. Additionally, an obvious difference was observed between the patients with a lower and higher PNI value in the serum white blood cell, hemoglobin, and red blood cell counts. Patients in the lower PNI group were also more likely to have more frequent postoperative complications, more blood loss, a longer surgery time and a longer hospital stay.

**Table 3:** Clinicopathological correlations of patients classified by PNI

Variables	PNI<45.6 (n=156)	PNI≥45.6 (n=216)	*p* value
Gender (male/female)	112/44	186/30	**0.001**
Age (y), mean±SD	52.29±11.00	51.35± 10.81	0.561
TBIL (μmol/L)	38.03± 77.05	19.51± 24.97	**0.044**
AST (U/L), mean±SD	62.01± 57.82	42.30± 44.19	**0.009**
ALT (U/L), mean±SD	56.40± 50.04	49.85± 59.25	0.429
ALB (g/dl), mean±SD	34.59± 4.47	42.61± 4.06	**<0.001**
ALBI	-2.06± 0.41	-2.84± 0.33	**<0.001**
RBC (10^12^/L), mean±SD	3.88± 0.55	4.48 0±.51	**<0.001**
Hb (g/dl), mean±SD	120.11± 21.10	138.75± 17.11	**<0.001**
PLT (10^9^/L), mean±SD	107.02± 72.47	135.18± 53.36	**0.003**
WBC (10^9^/L), mean±SD	4.79± 2.82	5.58± 2.25	**0.034**
PT (s), mean±SD	13.92± 1.64	13.22± 1.06	**0.001**
APTT (s)	39.20± 8.41	36.4± 5.79	**0.015**
INR (g/l), mean±SD	1.14± 0.14	1.05± 0.09	**<0.001**
AFP (ng/ ml), mean±SD	1981.6± 456.0	1857.1± 444.8	0.897
Number of tumours			
Single	126	196	**0.005**
Multiple	30	20	
Tumor size (cm), mean±SD	3.57± 1.11	3.51± 0.99	0.708
Surgery time (min)	207.76 ±75.48	199.53± 74.80	0.462
Blood loss (ml)	848.07± 880.79	463.08± 388.29	**<0.001**
Pringles maneuver time (min)	11.66± 13.64	11.36± 14.19	**0.883**
Length of hospital stay (day)	23.23± 8.36	20.44± 7.22	**0.016**
Overall Complication rate	104/156	62/216	**<0.001**
Major Complication rate	16/156	10/216	**0.036**

Abbreviations: TBIL, total bilirubin; AST, aspartate aminotransferase; ALT, alanine aminotransferase; ALB, albumin; ALBI, albumin-bilirubin score; RBC, red blood cell count; Hb, hemoglobin; PLT, platelet count; WBC, white blood cell count; PT, prothrombin time; APTT, activated partial thromboplastin time; INR, international normalized ratio; AFP, alpha-fetoprotein; bold values represent *p* values less than 0.05.

**Figure 3:**
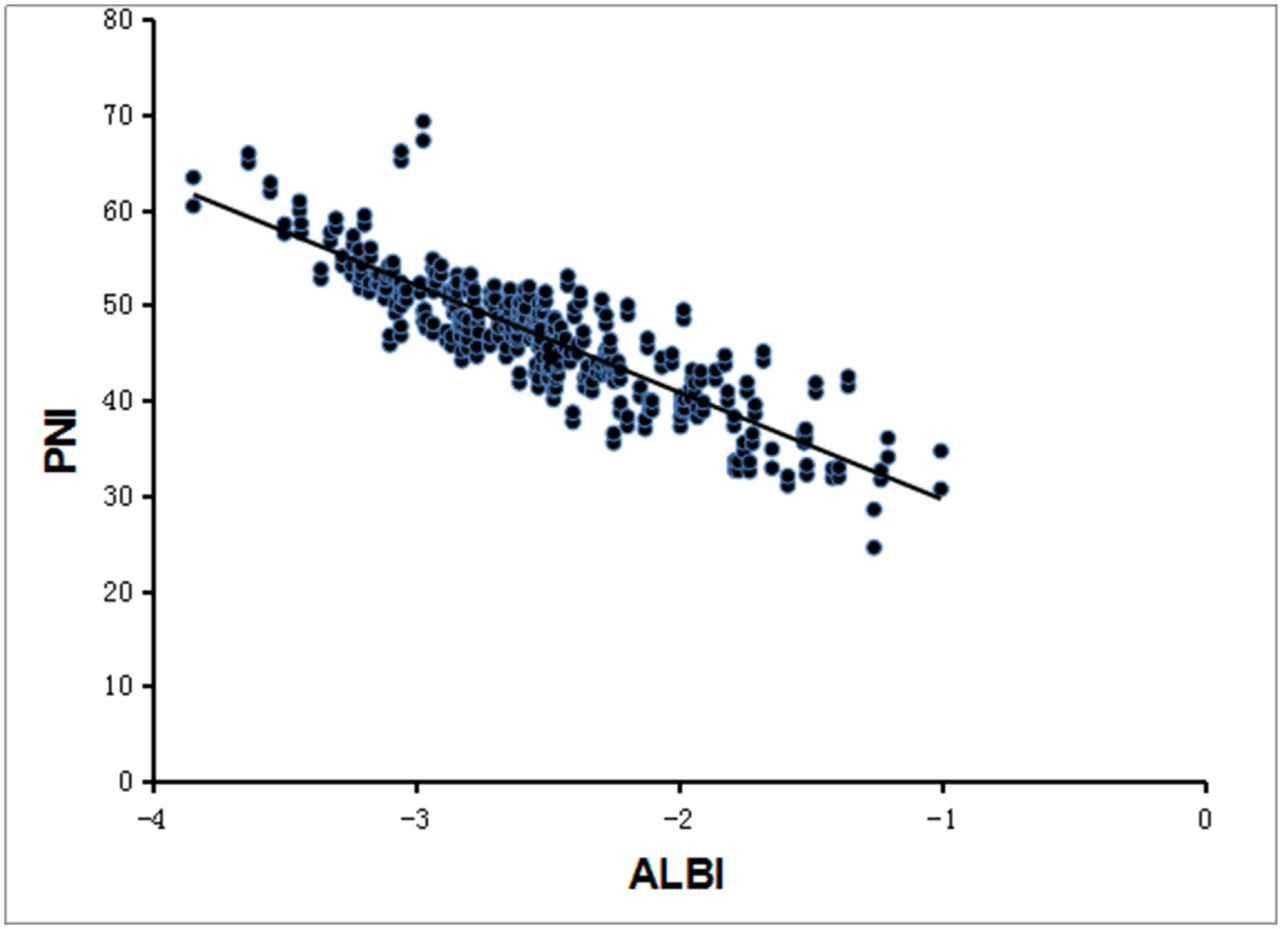
Correlation between PNI and ALBI The preoperative PNI and ALBI had a significant correlation by regression analysis (Pearson correlation coefficient, -0.865, *p* < 0.001).

**Figure 4:**
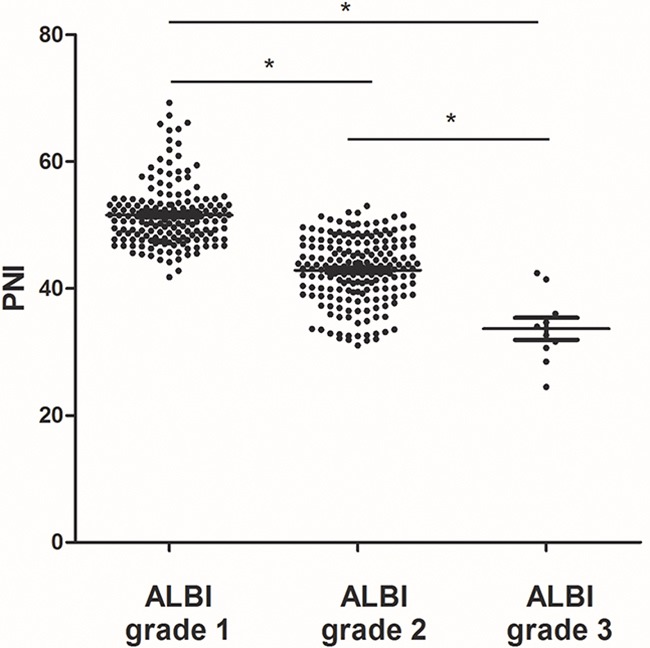
PNI levels in HCC patients with different ALBI grades Significant differences between the groups are marked by asterisks (*p < 0.001). The horizontal lines within the data signify the means.

Recently, the ALBIscore was validated as a simple, objective, and discriminatory method of assessing liver function. We found that there was a significant correlation between the ALBI score and the PNI value (Pearson correlation coefficient, -0.865, *p* < 0.001; Figure 3). The PNI value had significant differences in different ALBI grading (grade 1 median 51.14, range 41.80-69.25; grade 2 median 43.15, range 31.02-53.01; and grade 3 median 33.31, range 24.50-42.45, respectively; *p* < 0.001; Figure 4).

### Univariate and multivariate analyses

Postoperative complications occurred in 166 (44.6 %) patients in total; 104 patients had a PNI < 45.6, and 62 patients had a PNI ≥ 45.6. In the univariate analysis, PNI < 45.6, American Society of Anesthesiologists (ASA) grading, white blood cell count (WBC), platelet count, albumin, AST, PT, INR, operation time, bleeding and Pringle's maneuver time were closely related to postoperative complications. Additionally, multivariate analysis found that the WBC (HR, 0.824; 95% CI, 0.700-0.970; *p* = 0.020), a PNI < 45.6 (HR, 0.372; 95% CI, 0.180-0.766; *p* = 0.005), bleeding (HR, 1.002; 95% CI, 1.001–1.003; *p* < 0.001), and INR (HR, 3.091; 95% CI, 1.823–5.618; *p* = 0.007) were independently associated with the postoperative complications' incidence (Table 4).

**Table 4:** Univariate and multivariate logistic regression analyses of postoperative complications

Variables	No complications	Complications	Univariate analysis	Multivariate analysis	
	(n=206)	(n=166)	*P* value	Hazard ratio (95% CI)	*p* value
Gender (male/female)	166/40	132/34	0.798		
Age (years)	50.78± 11.27	52.93±10.30	0.180		
ASA grading	2.19± 0.56	2.39± 0.62	**0.022**		
RBC (10^12^/L)	4.27± 0.60	4.17± 0.61	0.302		
Hb (g/dl)	132.91± 20.95	128.49± 20.86	0.154		
WBC (10^9^/L)	5.67± 2.72	4.73± 2.17	**0.011**	0.824 (0.700-0.970)	**0.020**
PLT (10^9^/L)	132.52± 66.63	112.02± 57.68	**0.028**		
TBIL (μmol/L)	21.11±42.94	34.94±64.63	0.096		
ALB (g/L)	40.84± 5.30	37.27 ±5.80	**<0.001**		
ALT (U/L)	46.61 ±54.64	60.02 ±56.04	0.102		
AST (U/L)	42.86±45.40	60.13±56.31	**0.025**		
PT (s)	13.18± 1.11	13.93± 1.55	**<0.001**		
APTT (s)	37.74± 7.65	37.45± 6.44	0.781		
INR	1.05± 0.09	1.14± 0.14	**<0.001**	3.091 (1.823–5.618)	0.007
Tumor number	1.11± 0.35	1.25± 0.64	0.084		
Tumor size (cm)	3.51± 1.06	3.56± 1.02	0.774		
PNI <45.6 vs. ≥45.6	52/154	104/62	**<0.001**	0.372 (0.180-0.766)	**0.005**
Blood loss (ml)	389.70± 280.32	915.06± 868.40	**<0.001**	1.002 (1.001-1.003)	**<0.001**
Operation time (min)	190.97± 72.14	217.90± 76.21	**0.015**		
Pringles maneuver time	9.66± 14.53	13.75±12.87	**0.046**		

Abbreviations: ASA, American Society of Anesthesiologists; RBC, red blood cell count; Hb, hemoglobin; WBC, white blood cell count; PLT, platelet count; TBIL, total bilirubin; ALB, albumin; ALT, alanine aminotransferase; AST, aspartate aminotransferase; PT, prothrombin time; APTT, activated partial thromboplastin time; INR, international normalized ratio; PNI, prognostic nutritional index; bold values represent **p** values less than 0.05.

## DISCUSSION

As the liver is an important metabolic organ, liver cancer has been associated with increased malnutrition rates. It is well-known that nutritional status is closely associated with short- and long-term outcomes, such as the length of hospital stay, the rate of postoperative complications, disease-free survival and overall survival [8, 9]. Furthermore, alterations of metabolic and immune systems are tightly related to high rates of postoperative morbidity.

In the clinical practice, the PNI value, a combination of the albumin and total lymphocyte count, was initially used to evaluate the immunological and nutritional aspects of patients undergoing surgery of the gastrointestinal tract [10–12]. Prior studies have shown that the PNI can predict long-term prognosis. For example, it has been found that the PNI was an independent factor of poor survival for HCC patients [10, 13]. In addition, several studies have determined that low PNI has been significantly related to the rate of postoperative complications. It has also been reported that preoperative PNI was a useful index to predict postoperative complications in colorectal cancer patients with high-grade [14]. Meanwhile, scientists exhibited that preoperative PNI can be used to identify patients who have increased risk of postoperative complications [5].

In this paper, we found that the PNI correlated tightly with the hepatic function index, the parameters of coagulation function, and the white blood cell, hemoglobin, and red blood cell counts. In the lower PNI group, patients were also more likely to have postoperative complications, more blood loss, and a longer surgery time and hospital stay. Multivariate analysis revealed that PNI was an independent factor for predicting complications after operation. We all know that major liver resection carries a high risk for postoperative complications. In the current study, we eliminated patients who would require these major liver resections by excluding patients with HCC beyond the Milan criteria. In the studied population, we showed that the preoperative PNI could be used to predict postoperative complications with moderate accuracy.

So far, the mechanism of how a low PNI value leads to a worse prognosis is unclear. It is well known that malnutrition is a factor tightly correlated with the incidence of postoperative complications. Previous studies have demonstrated that perioperative nutrition has been not only a tool to supply calorie and nitrogen support but also a therapeutic strategy aimed at enhancing the immune system and increasing resistance to complications. Given that PNI was initially constructed as a reflection of a patient's nutritional status, it makes sense that the PNI may be related to postoperative complications. Our results suggest that the PNI is an independent risk predictor for postoperative complications. This result is consistent with our hypothesis as well as several previous studies about evaluating the predictive role of the PNI in postoperative complications [5, 14].

The PNI includes the lymphocyte count in its calculation. Wada et al. found that inflammatory cell infiltration of tumors is associated with a better prognosis in HCC patients due to the antitumor effect induced by the cellular immunity of T lymphocytes [15]. Additionally, it has also been found that the level of serum albumin and the count of lymphocyte had a tight relationship with the induction of the inflammatory response [16]. Therefore, it not only reflects the status of nutrition but also systemic inflammation. For patients with a low PNI, clinicians can try to improve their outcomes through perioperative nutritional interventions, for example, the administration of branched-chain amino acid-enriched nutrient support [17, 18].

As a new index, the ALBI is better than the Childs-Pugh score at reflecting liver function. Although the PNI and the ALBI both include serum albumin in their calculation, their formulas are different. Since the relativity analysis showed that the PNI was significant correlated with the ALBI, the PNI may also be an indicator of liver function, which can predict postoperative complications.

However, there are several limitations in our study. This study was retrospective, so the retrospective nature of the analysis may affect the results of this study. Further prospective studies are needed to confirm and update our conclusion. In our study, although the PNI achieved good predictive accuracy, it had a 38.5% and a 30.4% false-positive rate and false-negative rate, respectively, in predicting postoperative complications. This remains high if major clinical decisions are needed. Another limitation in the present study may be the lack of preoperative nutritional status evaluations. Some nutritional risk assessment tools, such as the Malnutrition Universal Screening Tool, Nutritional Risk Index and Nutritional Risk Screening 2002, can evaluate the nutritional state of patients. It has been shown that a higher Nutritional Risk Score has been related to adverse postoperative outcomes. Nevertheless, the PNI has advantages over these tools that cannot be ignored: the PNI was developed based on statistical evidence rather than clinical observation. Moreover, the PNI, including two commonly measured variables (serum albumin and lymphocytes), is evaluated objectively. Our study adds to the evidence that the PNI plays an important role in assessing the risk of short-term post-operative complications in patients with HCC after liver resection.

In conclusion, our results suggest that the PNI is a useful tool in assessing the postoperative morbidity in HCC patients after hepatectomy. The PNI is simple to calculate from laboratory measures. It is cheap, easily determined, and reproducible. Therefore, the PNI should be further evaluated as a prognostic marker to predict the risk of postoperative complications in HCC patients at diagnosis, who are to undergo liver resection.

## MATERIALS AND METHODS

### Patient population and data collection

Three-hundred and seventy-two patients treated with partial hepatectomies for HCC within the Milan criteria (solitary nodule ≤ 5 cm; ≤ 3 nodules, none > 3 cm; and no macrovascular invasion) were identified from The First Affiliated Hospital of Xi'an Jiaotong University between January 1, 2003, and December 31, 2014. All patients provided informed consent, and the study was approved by the ethics committee of Xi'an Jiaotong University. The preoperative diagnosis was based on the criteria of the American Association for the Study of Liver Diseases [19]. Patients with a positive history of inflammatory disease, infection, exposure to steroids at the time of diagnosis, and those who subsequently were diagnosed with non-HCC malignancy after histopathologic examinations were excluded. Standard demographic and clinicopathological data were collected, including the following: age, sex, body mass index (BMI), routine blood count, liver and kidney functions, alpha fetal protein (AFP), tumor focus, and complete blood count. These were routinely obtained in all patients within one week before surgery.

The PNI was computed as follows: serum albumin (g/L) + 5 × lymphocyte count (10^9^/L) [13]. ALBI score was calculated by the formula, −0.085 × (albumin g/l) + 0.66 × l0g_10_ (bilirubin μmol/l), and the patients were stratified into three groups: ALBI grade 1 (≤−2.60), grade 2 (>−2.60 to −1.39) and grade 3 (>−1.39) [20]. Perioperative morbidity and mortality included complications or death during the hospital stay or within 30 days of operation. Complications were categorized into 7 grades according to the modified Clavien classification [21].

### Statistical methods

The continuous variables were compared with Student's *t* test or the Mann-Whitney U test, as appropriate, whereas categorical variables were compared with the χ^2^ test or Fisher exact test, as appropriate. The diagnostic performance of PNI for predicting postoperative complications was assessed by the ROC curve and the AUROC. The optimal cutoff value was set as the value with the highest sum of specificity plus sensitivity. Multivariate analysis for the incidence of postoperative complications was carried out with binomial logistic regression.

SPSS 20.0 software (SPSS Inc., Chicago, Illinois, USA) was used to carry out all the statistical analyses. All tests were 2-sided, and *p* value < 0.05 was regarded as statistically significant.
